# Comment on “Ebola Virus Infection among Western Healthcare Workers Unable to Recall the Transmission Route”

**DOI:** 10.1155/2017/7458242

**Published:** 2017-09-20

**Authors:** Mark G. Kortepeter, Theodore J. Cieslak, Elena H. Kwon, Philip W. Smith, Christopher J. Kratochvil, Angela L. Hewlett

**Affiliations:** ^1^Medical Division, Laulima Government Solutions, Orlando, FL, USA; ^2^US Army Medical Research Institute of Infectious Diseases (USAMRIID), Fort Detrick, MD, USA; ^3^College of Public Health, University of Nebraska, Omaha, NE, USA; ^4^Nebraska Biocontainment Unit and College of Public Health, University of Nebraska, Omaha, NE, USA; ^5^Medical Division, USAMRIID, 1425 Porter Street, Fort Detrick, MD 21702, USA; ^6^College of Public Health, University of Nebraska Medical Center, Omaha, NE, USA; ^7^University of Nebraska Medical Center, Omaha, NE, USA; ^8^Nebraska Medicine, Omaha, NE, USA; ^9^UNeHealth, Omaha, NE, USA; ^10^Division of Infectious Diseases and Nebraska Biocontainment Unit, University of Nebraska Medical Center, Omaha, NE, USA

We read with interest the recent paper by Petti et al. entitled “Ebola Virus Infection among Western Healthcare Workers Unable to Recall the Transmission Route” [1]. Although the article relies on prior reports from healthcare organizations or magazine and newspaper articles rather than direct interviews with the patients themselves, we still believe the article potentially validates some of our concerns related to caring for patients infected with Ebola virus.

During the 2014–16 Ebola virus outbreak in West Africa, healthcare workers were at greater risk of infection than the population at large [2]. The African healthcare environment is much less controlled than a developed-world healthcare facility; however, as noted by Petti et al. [1], in both developed and underdeveloped settings, even contacts deemed to be low risk ended up becoming infected. Without an easily identifiable breach in personal protective measures, such as a recognized mucosal splash or needle stick, it is impossible to pinpoint definitively the exact moment or mechanism of exposure.

We still have much to learn regarding potential exposure mechanisms and exposure routes in the clinical setting. Therefore, as we have argued previously, there is no room for error with a disease like Ebola that leads to the production of copious amounts of body fluids with high viral titers, along with the usual challenges of following infection control and safety protocols to the letter in any healthcare setting [3]. This is why we continue to advocate for the utilization of high-level containment care units, when such facilities are available, for viral hemorrhagic fevers that have demonstrated infection of healthcare workers, like Ebola, Marburg, Crimean Congo hemorrhagic fever, and Lassa viruses, and potentially others [4]. This option protects caregivers through sophisticated engineering controls (directional airflow, hot/cold designations to identify graduated infection risk, and pass-through autoclaves), restricted access, staff who are well-practiced in donning and doffing protocols, and other unique infection control practices needed to limit spread to healthcare workers [5].
